# Current management of cancer-associated venous thromboembolism in patients with thrombocytopenia: a retrospective cohort study

**DOI:** 10.1007/s11739-021-02771-3

**Published:** 2021-06-10

**Authors:** Alessandro Squizzato, Silvia Galliazzo, Elena Rancan, Marina Di Pilla, Giorgia Micucci, Gianmarco Podda, Emanuele Valeriani, Leonardo Campiotti, Lorenza Bertù, Walter Ageno, Ettore Porreca, Corrado Lodigiani

**Affiliations:** 1 Research Center On Thromboembolic Disorders and Antithrombotic Therapies, University of Insubria, Varese and Como, Italy; 2 Department of Internal Medicine, San Valentino Hospital, Montebelluna (Treviso), Italy; 3 Thrombosis and Hemorragic Diseases Unit, IRCCS Humanitas Clinical and Research Hospital, Milan, Italy; 4 Clinic of Hematology, Ancona University, Ancona, Italy; 5 U.O. Medicina III, Department of Health Science, ASST Santi Paolo e Carlo-University of Milan, Milan, Italy; 6 Department of Medical, Oral and Biotechnological Sciences “G. D’Annunzio” University, Chieti, Italy; 7 U.O.C. Medicina, ULSS 2, Ospedale San Valentino, via Palmiro Togliatti 1, 31044 Montebelluna (Treviso), Italy

**Keywords:** Thrombocytopenia, Cancer associated thrombosis, Anticoagulation

## Abstract

Optimal management of venous thromboembolism (VTE) in cancer patients with thrombocytopenia is uncertain. We described current management and clinical outcomes of these patients. We retrospectively included a cohort of cancer patients with acute VTE and concomitant mild (platelet count 100,000–150,000/mm^3^), moderate (50,000–99,000/mm^3^), or severe thrombocytopenia (< 50,000/mm^3^). Univariate and multivariate logistic regression analyses explored the association between different therapeutic strategies and thrombocytopenia. The incidence of VTE and bleeding complications was collected at a 3-month follow-up. A total of 194 patients of whom 122 (62.89%) had mild, 51 (26.29%) moderate, and 22 (11.34%) severe thrombocytopenia were involved. At VTE diagnosis, a full therapeutic dose of LMWH was administered in 79.3, 62.8 and 4.6% of patients, respectively. Moderate (OR 0.30; 95% CI 0.12–0.75), severe thrombocytopenia (OR 0.01; 95% CI 0.00–0.08), and the presence of cerebral metastasis (OR 0.06; 95% CI 0.01–0.30) were independently associated with the prescription of subtherapeutic LMWH doses. Symptomatic VTE (OR 4.46; 95% CI 1.85–10.80) and pulmonary embolism (OR 2.76; 95% CI 1.09–6.94) were associated with the prescription of full therapeutic LMWH doses. Three-month incidence of VTE was 3.9% (95% CI 1.3–10.1), 8.5% (95% CI 2.8–21.3), 0% (95% CI 0.0–20.0) in patients with mild, moderate, and severe thrombocytopenia, respectively. The corresponding values for major bleeding and mortality were 1.9% (95% CI 0.3–7.4), 6.4% (95% CI 1.7–18.6), 0% (95% CI 0.0–20.0) and 9.6% (95% CI 5.0–17.4), 48.2% (95% CI 16.1–42.9), 20% (95% CI 6.6–44.3). In the absence of sound evidence, anticoagulation strategy of VTE in cancer patients with thrombocytopenia was tailored on an individual basis, taking into account not only the platelet count but also VTE presentation and the presence of cerebral metastasis.

## Introduction

Venous thromboembolism (VTE) threatens the clinical course of cancer, representing a strong predictor of decreased survival for all cancer types [1]. Despite the recommended anticoagulant treatment, cancer-associated thrombosis (CAT) is linked to high risk of major bleeding and recurrent thrombosis [2]. The treatment of CAT becomes even more challenging in the presence of thrombocytopenia. Cancer-associated thrombocytopenia may be transient or persistent, as many pathophysiological mechanisms may be involved, such as chemotherapy, metastatic bone marrow infiltration and disseminated intravascular coagulation. Moreover, cancer-associated thrombocytopenia has a wide variable severity, ranging from mild (between 100,000 and 150,000/mm^3^) to a very severe decrease in platelet count (less than 20,000/mm^3^).

Treatment with low-molecular weight heparin (LMWH) has been the standard of care for CAT for many years, but little is known about the optimal treatment in patients at higher risk of bleeding, such as those with thrombocytopenia. Since low platelet count is usually an exclusion criterion in studies of anticoagulant drugs, patients with a platelet count lower than 75,000/mm^3^ or 50,000/mm^3^ were excluded from all clinical trials on CAT management [2, 3]. Moreover, the occurrence of thrombocytopenia has been associated not only with increased bleeding risk, but also with a higher risk of recurrent VTE [3, 4].

The optimal management of CAT in patients with low platelet count remains a clinically relevant unmet need in the absence of committed, randomized controlled studies. Case series suggest a highly heterogeneous approach to these scenarios in current clinical practice [5–16]. Some experts attempted to address this topic by providing guidance for anticoagulation therapy on the basis of platelet count [17]. Unfortunately, this guidance provides recommendations based only on platelet levels and a risk estimation of thrombus progression. We believe that other clinical variables should be taken into account to optimize CAT treatment. Actually, the role of platelet count should be carefully weighed with that of common and cancer-specific thrombotic and haemorrhagic risk factors, such as brain metastasis. In the current study we have sought to evaluate, in a cohort of patients with CAT and thrombocytopenia: (i) treatment strategies used by experts in thrombosis and hemostasis to manage CAT, (ii) potential factors associated with diverse treatment approaches, (iii) the 3-month incidence of recurrent VTE, major bleeding, and mortality.

## Methods

This study is designed as a multicentric retrospective cohort study of patients with acute CAT and concomitant thrombocytopenia who were referred to five Italian academic tertiary care Thrombosis Centers (Insubria University Hospital, Varese; IRCCS Humanitas Research Hospital, Milan; G. d’Annunzio University of Chieti-Pescara; Milan University Hospital and Ancona University Hospital) from 2006 to 2018. The Medical Ethical Committee of Varese approved protocol on the 27th of Sep 2016.

### Study population

A cohort of adult patients with active cancer and VTE with concomitant thrombocytopenia at VTE diagnosis was identified by retrospectively querying the electronic database of each Thrombosis Center.

VTE included symptomatic or incidentally detected VTE and was objectively confirmed by imaging tests. The diagnosis of pulmonary embolism was made in the presence of a positive computed tomographic pulmonary angiography (CTPA), V/Q lung scan or perfusion lung scan with negative chest X-ray. Objective diagnosis of proximal or distal deep vein thrombosis (DVT) was made by compression ultrasonography. We considered both lower and upper limb DVT and also included symptomatic or incidental splanchnic vein thrombosis (i.e., Budd–Chiari syndrome, portal vein thrombosis, mesenteric vein thrombosis, and splenic vein thrombosis) detected by abdominal ultrasound or computed tomography. Incidentally detected VTE was diagnosed via an imaging test performed for other clinical indications (e.g., cancer staging or follow-up). Thrombocytopenia was defined as a platelet count lower than 150 000/mm^3^ at VTE diagnosis.

We included active solid and haematological cancer (e.g., leukaemia, multiple myeloma, lymphoma, myeloproliferative, and myelodysplastic disorders). Active cancer was defined as follows: (i) cancer diagnosis within 6 months before study inclusion, (ii) ongoing treatment for cancer at the time of inclusion or any treatment for cancer during the previous 6 months, or (iii) recurrent, locally advanced or metastatic disease (for solid tumor), or not in complete remission (for haematological malignancy). No exclusion criteria were applied.

### Outcomes

The primary outcome was the dosage of anticoagulant therapy used in patients with mild, moderate, and severe thrombocytopenia. We considered as intermediate dose the 75% of the LMWH therapeutic dose. We defined as an escalation of LMWH dose the switch from the prophylactic to the intermediate dose or the switch from the intermediate to the therapeutic dose. The opposite switch was defined as a reduction of LMWH dosage. Thrombocytopenia was defined mild when the platelet count was between 100,000 and 149,000/mm^3^, moderate for a platelet count between 50,000 and 99,000/mm^3^, and severe for a platelet count < 50,000/mm^3^.

Secondary outcomes were the type and length of anticoagulant therapy in patients with mild, moderate, and severe thrombocytopenia. Secondary clinical outcomes were VTE recurrence, bleeding, and overall mortality at 3 months in patients with mild, moderate, and severe thrombocytopenia. According to International Society of Thrombosis and Haemostasis (ISTH) criteria, a major bleeding was defined as an overt bleeding that was associated with a decreased in the hemoglobin level of 2 g/dL or more, led to a transfusion of two or more units of blood, occurred in a critical site, or contributed to death. [18]. Clinically relevant non-major bleedings (CRNMB) were defined as any non-major bleedings that have clinical consequences for a patient such as medical intervention, the need for an unscheduled contact with a physician or temporary cessation of anticoagulant treatment or associated with pain or impairment of daily activities. All the other non-major bleedings that did not meet the criteria for CRNMB were defined as minor bleedings.

In addition, all outcomes were analyzed according to patient’s age, type of cancer (hematological vs solid cancer), type of VTE index event, clinical presentation (symptomatic vs incidentally-diagnosed VTE), presence of metastasis, presence of central venous catheter and ongoing chemotherapy. We considered as symptomatic all VTE events that were suspected on clinical grounds and then confirmed by means of diagnostic imaging.

Patients were treated and followed up in the primary centers where diagnosis of VTE was made. All outcomes were locally assessed by the treating physician.

#### Data extraction

We abstracted the following patient baseline characteristics at VTE diagnosis: age, sex, type and site of active cancer, presence of metastasis, ongoing chemotherapy, presence of central venous catheter, and platelet count. For all patients, we reported the anticoagulant strategy adopted by the attending physician, the use of any platelet transfusions, and the insertion of inferior vena cava filter. Any change in anticoagulation treatment (e.g., LMWH dose escalation, reduction, or withdrawal) since VTE diagnosis was recorded using information from follow-up visits and clinical notes.

### Statistical analysis

Few data are available in the literature on this topic. A recent retrospective study suggested that 23% of patients with thrombocytopenia (a platelet count lower than 50,000/mm^3^) with CAT did not receive any anticoagulant therapy [12].

By hypothesizing a similar proportion of untreated patients with severe thrombocytopenia and < 5% of untreated patients among those with mild thrombocytopenia, a total of at least 136 patients should be included to show a statistically significant difference (*p* < 0.05) between treatment strategies (i.e., percentage of patients with mild or severe thrombocytopenia in whom anticoagulant treatment is withdrawn), with an *α* error of 0.05 and a statistical power (1−β error) of 80%.

Data were described as mean and standard deviation (SD) for continuous variables and proportions for categorical variables. Two classifications for thrombocytopenia were adopted: mild (between 100,000 and 149,000/mm^3^), moderate (50,000 and 99,000/mm^3^), and severe (< 50,000/mm^3^); dichotomous classification (< 75,000/mm^3^ vs. ≥ 75,000/mm^3^; < 50,000/mm^3^ vs ≥ 50,000/mm^3^). These cut-offs were adopted in the attempt to fill the knowledge gap, as patients with a platelet count lower than 75,000 and 50,000/mm^3^ were excluded or were only a tiny minority in the published trials on CAT management [2, 3].

Univariate and multivariate logistic regression analyses were used to explore the association between the therapeutic strategies and the following variables: platelet count, age, presence of metastases, ongoing chemotherapy, type of cancer (solid or hematologic), and VTE index event (PE or no PE, in this latter group we considered all DVT without PE).

Two tailed *p *values < 0.05 were used to indicate statistical significance.

All analyses were carried out using the SAS statistical package (Version 9.4 for Windows. SAS Institute Inc. Cary NC).

## Results

A total of 194 patients were included in our cohort study. Baseline characteristics are detailed in Table 1. The median age was 64.9 ± 11.4 years, and 108 patients (55.7%) were male. One hundred and forty-four patients (144/194, 74.2%) had solid cancer which was metastatic in 91 (91/194, 46.9%), with cerebral metastasis in 16 (16/194, 8.2%). One hundred and twenty-nine patients (129/194, 66.5%) were on active chemotherapy and VTE was symptomatic in 144 (144/194, 74.2%). Sites of venous thrombotic events are detailed in Table 1. Thrombocytopenia at VTE diagnosis was mild in 121 patients (121/194, 62.4%), moderate in 51 (51/194, 26.3%), and severe in 22 (22/194, 11.3%), including five with a platelet count lower than 20,000/mm^3^. One hundred and forty-six patients (146/194, 75.3%) had a platelet count greater than 75,000/mm^3^ while the remaining forty-eight (48/194, 24.7%) had a platelet count lower than 75,000/mm^3^.

**Table 1:** Baseline characteristics of study patients, *N* = 194

	Total population (*n* = 194)	Mild thrombocytopenia (*n* = 121)	Moderate thrombocytopenia (*n* = 51)	Severe thrombocytopenia (*n* = 22)
Age, median (SD)	64.9 ± 11.4	66.0 (10.5)	65.5 (12.7)	58 (11.5)
Male, *n* (%)	108 (55.7)	69 (57.0)	27 (52.9)	12 (54.6)
Thrombocytopenia				
Mild, *n* (%)	121 (62.4)			
Moderate, *n* (%)	51 (26.3)			
Severe, *n* (%)	22 (11.3)			
Thrombocytopenia				
≥75,000, *n* (%)	147 (75.4)			
< 75,000, *n* (%)	48 (24.6)			
Symptomatic VTE, *n* (%)	144 (74.2)	92 (76.0)	38 (74.5)	14 (63.6)
VTE index event				
pulmonary embolism, *n* (%)	29 (14.4)	24 (19.8)	3 (5.9)	1 (4.6)
DVT + PE, *n* (%)	46 (23.7)	34 (28.1)	9 (17.6)	3 (13.6)
Lower extremities DVT, *n* (%)	57 (29.5)	35 (28.9)	19 (37.3)	3 (13.6)
Lower extremities isolated distal DVT, *n* (%)	4 (2.1)	3 (2.5)	1 (2.0)	0 (0.0)
Upper extremities DVT, *n* (%)	40 (20.6)	20 (16.5)	10 (19.6)	10 (45.5)
Lower and upper extremities SVT, *n* (%)	2 (1.0)	0 (0.0)	1 (2.0)	1 (4.6)
Splanchnic/inferior vena cava DVT, *n* (%)	18 (9.3%)	6 (5.0)	8 (15.7)	4 (18.2)
Cancer				
Haematologic neoplasm, *n* (%)	51 (26.3)	22 (18.2)	16 (31.4)	13 (59.1)
Solid neoplasm, *n* (%)	144 (74.2)	100 (82.6)	35 (68.6)	9 (40.9)
Metastatic cancer, *n* (%)	91 (46.9)	71 (58.7)	14 (27.5)	6 (27.3)
Cerebral metastasis, *n* (%)	16 (8.2)	14 (11.6)	0 (0.0)	2 (9.1)
Ongoing chemotherapy, *n* (%)	129 (66.5)	86 (71.1)	29 (56.6)	14 (63.6)

### VTE treatment

In the group of patients with mild thrombocytopenia, 96 (96/121, 79.3%) received a full therapeutic dose of LMWH, 12 (12/121, 9.9%) an intermediate dose of LMWH, 8 (8/121, 6.6%) a prophylactic dose, and 5 (5/121, 4.1%) oral anticoagulation using a vitamin K antagonist (*n* = 1) or apixaban (*n* = 4). An inferior vena cava filter was placed in nine patients (9/121, 7.4%). During the first month, 23 patients (23/121, 19.0%) modified their initial anticoagulant strategy as follows: for 10 patients (10/23, 43.5%) the LMWH dose was reduced, for 1 (1/23, 4.3%) the LMWH dose was escalated, for 6 (6/23, 26.0%) there was a switch to warfarin, for 3 (2/23, 13.0%) there was a switch to fondaparinux, and for 3 (3/23, 13.0%) anticoagulation treatment was stopped. After the first month, 51 patients (51/121, 42.2%) were on a full LMWH dose, 44 (44/121, 36.4%) were on an intermediate LMWH dose, 9 (9/121, 7.4%) were on a prophylactic LMWH dose and 4 (4/121, 3.3%) were on oral anticoagulant. Within the first month 11 patients (11/121, 9.1%) were missing and 2 (2/121, 1.7%) died. In the group with moderate thrombocytopenia, 32 patients (32/51, 62.8%) received a full therapeutic LMWH dose, 13 (13/51, 25.5%) an intermediate LMWH dose, 3 (3/51, 5.9%) a prophylactic LMWH dose, and 2 (2/51, 3.9%) a direct oral anticoagulant. An inferior vena cava filter was placed in two patients (2/51, 3.9%), and platelets were transfused in 4 (4/51, 7.8%). During the first month, 18 patients (18/51, 35.2%) modified their initial anticoagulant strategy as follows: in nine patients the LMWH dose was reduced, in two the LMWH dose was escalated, in one there was a switch to warfarin, in one there was a switch to fondaparinux, in one there was a switch to direct oral anticoagulant and in four anticoagulation was stopped. After the first month, 24 patients (24/51, 47.1%) were on a LMWH full dose, 12 (12/51, 23.5%) were on an intermediate LMWH dose, 4 (4/51, 7.8%) were on a prophylactic LMWH dose, 2 (2/51, 4.0%) were on an oral anticoagulant, and 6 (6/51, 11.8) did not receive anticoagulation. Within the first month two patients (2/51, 3.9%) were missing and one patient (1/51, 2.0%) died.

In the group with severe thrombocytopenia, one patient (1/22, 4.6%) received a full LMWH therapeutic dose, 10 (10/22, 45.5%) an intermediate LMWH dose, 5 (5/22, 22.7%) a prophylactic LMWH dose, 1 (1/22, 4.5%) received low dose of apixaban (2.5 mg bid), and 5 (5/22, 22.7%) received no anticoagulation. An inferior vena cava filter was placed in two patients (9.1%) and platelets were transfused in 13 (59.1%). During the first month, two patients (2/22, 9.1%) required an LMWH dose reduction, 8 (8/22, 36.4%) an LMWH dose escalation, and 2 (2/22, 9.1%) stopped anticoagulation treatment. After the first month, two patients (2/22, 9.1%) were on a full LMWH dose, 7 (7/22, 31.8%) were on an intermediate LMWH dose, 4 (4/22, 18.2%) were on a prophylactic LMWH dose, 4 (4/22, 18.2%) did not receive anticoagulation, 1 (1/22, 4.5%) were on oral anticoagulant. Within the first month two patients (2/22, 9.1%) were missing and two patients (2/22, 9.1%) died.

According to multivariate analysis, the variables independently associated with a subtherapeutic LMWH dose (e.g., an intermediate or prophylactic dose) or no anticoagulation were both moderate and severe thrombocytopenia (OR = 0.30; 95% CI 0.12–0.75 and OR = 0.014; 95% CI 0.003–0.083, respectively) as well as the presence of cerebral metastasis (OR = 0.06; 95% CI 0.01–0.30). Conversely, a symptomatic VTE and a diagnosis of PE were related to the choice of a full therapeutic dose of LMWH (OR = 4.46; 95% CI 1.85–10.80; and OR = 2.76; 95% CI 1.09–6.94, respectively) (Table 2). When the multivariate analysis was repeated by including the dichotomous classification of thrombocytopenia around 50,000 and 75,000/mm^3^, we found that a platelet count lower than the pre-specified cut-off was associated with no anticoagulant therapy or treatment with a subtherapeutic LMWH dose (e.g., an intermediate or prophylactic dose) (OR = 0.03; 95% CI 0.01–0.13 and OR = 0.09; 95% CI 0.04–0.23, respectively). All the other associations previously described were reconfirmed (Tables 3, 4).

**Table 2:** Univariate and multivariate predictors of anticoagulant strategy in the acute phase of VTE index event

		Univariate analysis		Multivariate analysis	
	*n*/ev	OR	CI 95%	OR	CI 95%
Thrombocytopenia					
Mild	121/101	1.0	Ref	1.0	Ref
Moderate	51/34	0.4	**0.19; 0.84**	0.30	0.12; 0.75
Severe	22/2	0.02	**0.004; 0.092**	0.014	0.003; 0.083
Age (years)					
< 65	86/56	1.0	Ref	1.0	Ref
≥ 65	108/81	1.61	0.86; 2.29	0.86	0.39–1.89
Metastasis					
No	103/71	1.0	Ref	1.0	Ref
Cerebral	16/8	0.45	0.16; 1.31	**0.06**	**0.01; 0.3**
Other than cerebral	75/58	1.54	0.78; 3.04	0.50	0.18; 1.38
Ongoing chemotherapy					
No	65/46	1.0	Ref	1.0	Ref
Yes	129/91	0.99	0.51; 1.90	0.85	0.36; 1.98
Type of cancer					
Solid	143/108	1.0	Ref	1.0	Ref
Haematologic	51/29	**0.43**	**0.22; 0.84**	0.39	0.13; 1.15
VTE index event					
No PE	122/78	1.0	Ref	1.0	Ref
PE	72/59	**2.56**	**1.27–5.18**	**2.76**	**1.09; 6.94**
Symptomatic VTE					
No	50/27	**1.0**	**Ref**	**1.0**	**Ref**
Yes	144/110	**2.8**	**1.4–5.4**	**4.46**	**1.85–10.8**

Bold values indicate OR and 95%CI significant for an association between the independent variable and the anticoagulant strategy

*ev* patients on LMWH full therapeutic dose

**Table 3:** Univariate and multivariate predictors of anticoagulant strategy in the acute phase of VTE index event

		Univariate		Multivariate	
	*n*/ev	OR	CI 95%	OR	CI 95%
Platelets count					
≥ 50,000	172/135	1.0	Ref	1.0	Ref
< 50,000	22/2	**0.03**	**0.01; 0.12**	**0.03**	**0.01; 0.13**
Age (years)					
< 65	86/56	1.0	Ref	1.0	Ref
≥ 65	108/81	1.61	0.86; 2.99	0.86	0.40; 1.89
Metastatic cancer					
No	103/71	1.0	Ref	1.0	Ref
Cerebral metastasis	16/8	0.45	0.16; 1.31	**0.11**	**0.03; 0.46**
No cerebral metastasis	75/58	1.54	0.78; 3.04	0.63	0.24; 1.65
Ongoing chemotherapy					
No	65/46	1.0	Ref	1.0	Ref
Yes	129/91	0.99	0.51; 1.90	1.03	0.45; 2.33
Cancer type					
Solid	143/108	1.0	Ref	1.0	Ref
Haematologic	51/29	**0.43**	**0.22; 0.84**	0.40	0.14; 1.15
VTE index event					
No PE	122/78	1.0	Ref	1.0	Ref
PE	72/59	**2.56**	**1.27; 5.18**	**3.33**	**1.35; 8.19**
Symptomatic VTE					
No	50/27	**1.0**	**Ref**	**1.0**	**Ref**
Yes	144/110	**2.8**	**1.4; 5.4**	**4.41**	**1.87–10.4**

Bold values indicate OR and 95%CI significant for an association between the independent variable and the anticoagulant strategy

*ev* patients on LMWH full therapeutic dose

**Table 4:** Univariate and multivariate predictors of anticoagulant strategy in the acute phase of VTE index event

		Univariate		Multivariate	
	*n*/ev	OR	CI 95%	OR	CI 95%
Platelets count					
≥ 75,000	146/121	1.0	Ref	1.0	Ref
< 75,000	48/16	**0.10**	**0.05; 0.21**	**0.09**	**0.04; 0.23**
Age (years)					
< 65	86/56	1.0	Ref	1.0	Ref
≥ 65	108/81	1.61	0.86; 2.99	0.86	0.40; 1.89
Metastatic cancer					
No	103/71	1.0	Ref	1.0	Ref
Cerebral metastasis	16/8	0.45	0.16; 1.31	**0.08**	**0.02; 0.34**
No cerebral metastasis	75/58	1.54	0.78; 3.04	0.51	0.19; 1.38
Ongoing chemotherapy					
No	65/46	1.0	Ref	1.0	Ref
Yes	129/91	0.99	0.51; 1.90	1.19	0.53; 2.67
Cancer type					
Solid	143/108	1.0	Ref	1.0	Ref
Haematologic	51/29	**0.43**	**0.22; 0.84**	0.48	0.17; 1.38
VTE index event					
No PE	122/78	1.0	Ref	1.0	Ref
PE	72/59	**2.56**	**1.27; 5.18**	**2.70**	**1.11; 6.58**
Symptomatic VTE					
No	50/27	**1.0**	**Ref**	**1.0**	**Ref**
Yes	144/110	**2.8**	**1.4; 5.4**	**4.35**	**1.84–10.31**

Bold values indicate OR and 95%CI significant for an association between the independent variable and the anticoagulant strategy

*ev* patients on LMWH full therapeutic dose

### Clinical outcomes

Twenty-three patients were lost in follow-up. Therefore, a clinical follow-up at 3 months was available for 171 patients (171/194, 88.1%) of whom 104 (104/171, 60.8%) initially had a mild thrombocytopenia, 47 (47/171, 27.5%) a moderate thrombocytopenia, and 20 (20/171, 11.7%) a severe thrombocytopenia. At 3 months, we recorded 4 (4/104, 3.9%) with recurrent VTE diagnosis, 2 (2/104, 1.9%) with major bleeding, and 10 deaths (10/104, 9.6%) in patients with mild thrombocytopenia. The corresponding numbers in patients with moderate thrombocytopenia were 4 (4/47, 8.5%), 3 (3/47, 6.4%), and 13 (13/47, 27.6%), respectively. In patients with severe thrombocytopenia, there were no recurrent VTE nor major bleeding events, and four deaths (4/20, 20%).

By splitting the study population on the basis of a platelet count of around 75,000/mm^3^, we reported 7 VTE recurrences (7/125, 5.6%), four major bleedings (4/125, 3.2%), and 12 deaths (12/125, 9.6%) in patients with a platelet count of ≥ 75,000/mm^3^. In patients with a platelet count of < 75,000/ mm^3^, we reported one VTE recurrence (1/46, 2.2%), one major bleeding (1/46, 2.2%), and 15 deaths (15/46, 32.6%) (Table 5).

**Table 5:** Outcomes during 3-month follow-up according to platelets count

	Thrombocytopenia						Thrombocytopenia		
3-months outcomes	Mild (*n* = 104)	Moderate (*n* = 47)	Severe (*n* = 20)	Mild vs moderate*	Mild vs severe*	Moderate vs severe*	≥ 75,000/mm3 (*n* = 125)	< 75,000/mm^3^ (*n *= 46)	≥ 75,000/mm^3^ vs < 75,000/mm^3^
Major bleeding	2 (1.9)	3 (6.4)	0 (0.0)	0.17	0.99	0.55	4 (3.2)	1 (2.2)	0.99
Minor bleeding	5 (4.8)	3 (6.4)	1 (5.0)	0.70	0.99	0.99	7 (5.6)	2 (4.4)	0.99
CRNMB	4 (3.9)	1 (2.1)	0 (0.0)	0.99	0.99	0.99	5 (4.0)	0 (0.0)	0.32
VTE recurrence	4 (3.9)	4 (8.5)	0 (0.0)	0.26	0.99	0.31	7 (5.6)	1 (2.2)	0.68
Death	10 (9.6)	13 (27.6)	4 (14.8)	0.004	0.24	0.51	12 (9.6)	15 (32.6)	0.0006
No Outcomes	75 (72.1)	21 (44.7)	15 (75.0)				84 (67.2)	27 (58.7)	
Missing	4 (3.9)	2 (4.2)	0 (0.0)				6 (4.8)	0 (0.0)	

*p value Chi-squared or Fisher’s exact test

## Discussion

Our retrospective cohort suggests that the optimal anticoagulation strategy of CAT in patients with concomitant thrombocytopenia is far from being standardized. In this setting, physicians choose the anticoagulant strategy by weighing different thrombotic and haemorrhagic risk factors, not just on the basis of platelet count. Bleeding and thrombotic risk appeared to be variable, while the short-term mortality was always high. Consistent with previous studies [5, 12], we found that clinicians adopted rather heterogeneous approaches, ranging from no treatment to full therapeutic dose anticoagulation. Intermediate doses of LMWH were preferred in about 30% of patients with moderate thrombocytopenia and in about 70% of patients with a platelet count below the 50,000/mm^3^ threshold. These patients had high short-term mortality, with nearly half of those with moderate thrombocytopenia dying within 3 months after VTE diagnosis. Rates of major bleeding and VTE recurrence, were numerically higher in patients with moderate thrombocytopenia compared to those with mild reduction in platelet count, but without any significant difference. Only all-cause mortality resulted significantly higher in patients with moderate compared to those with mild thrombocytopenia. Strikingly, among patients with severe thrombocytopenia there was no recurrent VTE nor major bleeding complications. It is very likely that the small size of the subgroup of patients with severe and moderate thrombocytopenia could have affected outcomes, thus impeding a clear explanation.

These findings confirm that managing the competing risks of thrombosis, bleeding, and anticoagulation is very complex. In our cohort, the approach used by clinicians was somewhat different from that suggested by ISTH experts [18]. It is interesting to note that multivariate analysis suggests that both low platelet count and the presence of cerebral metastasis were independently associated with a more cautious anticoagulation strategy. Conversely, a PE index event independently led clinicians to adopt a full LMWH therapeutic dose more frequently. Accordingly, based on clinical gestalt, the choice of anticoagulation strategy appears to rely on a multiparametric patient assessment encompassing common and cancer-specific thrombotic and bleeding risk factors.

Other issues need further discussion. First, the use of platelet transfusion was carried out in only a small minority of patients with moderate thrombocytopenia and in two-thirds of those with severe thrombocytopenia. Currently, ISTH experts suggest platelet transfusion as a prophylactic measure to maintain platelet count above 40,000–50,000/mm^3^ and to allow a full dose of anticoagulation, especially in patients considered at high risk of VTE recurrence [17]. Second, retrievable vein cava filter has been (unexpectedly) rarely inserted, probably due to little confidence in their use in this setting. The role of vein cava filter is a matter of discussion given the lack of proven evidence. However, it is theoretically a good option in patients at high bleeding risk, such as thrombocytopenic patients. Third, a close follow-up for this subset of CAT patients has proved essential to optimizing anticoagulant treatment during the first weeks after VTE diagnosis. Thrombotic and bleeding risk balance may rapidly change over time and, therefore, it should be dynamically reassessed to optimize CAT management. For instance, it is important to check platelet count trend (e.g., is the nadir or platelet count is expected to drop further?), as thrombocytopenia in these patients can be underpinned by various causes, both transient and permanent.

Our cohort investigated relevant variables that may influence physicians’ decision regarding anticoagulation treatment in one of the largest cohorts of CAT patients with thrombocytopenia. However, our result may be influenced by the retrospective design and their intrinsic potential biases, in particular the selection bias. Additional methodological limits include the relatively small group of CAT patients with severe thrombocytopenia and the lack of information available about thrombocytopenia duration as we ranked thrombocytopenia on the basis of the single measure of platelets count available at the time of VTE diagnosis. Information on data about details of metastasis, retrieval of vena cava filter, causes of death, sites of bleeding events, type of VTE recurrence and platelets count at the time of clinical outcomes are missing.

In conclusion, CAT patients with thrombocytopenia harbor many and various competing thrombotic and bleeding risk factors beyond low platelet count. The clinical decision-making process should be informed by a multiparametric assessment, not only on the basis of the degree of thrombocytopenia as suggested by some published guidelines. Further studies are needed to better investigate this challenging topic.

## Data Availability

The datasets generated during the current study are available from the corresponding author on reasonable request.
